# Stomal Outlet Obstruction Caused by Torsion of the Stomal Limb Associated with Fecal Loading after End Colostomy: A Case Report

**DOI:** 10.70352/scrj.cr.26-0337

**Published:** 2026-08-18

**Authors:** Sho Nambara, Koji Ando, Tetsuro Kawazoe, Shotaro Korehisa, Yasuo Tsuda, Tomonori Nakanoko, Eiji Oki, Tomoharu Yoshizumi

**Affiliations:** 1 Department of Surgery and Science, Graduate School of Medical Science, Kyushu University, Fukuoka, Fukuoka, Japan; 2 Department of Advanced Medicine and Innovative Technology, Kyushu University Hospital, Fukuoka, Fukuoka, Japan

**Keywords:** stomal outlet obstruction, torsion of the stomal limb, intraperitoneal route, constipation, elongated colon, splenic flexure mobilization

## Abstract

**INTRODUCTION:**

Stoma-related complications after colostomy include obstruction, prolapse, and parastomal hernia. However, torsion of the stomal limb associated with fecal loading in the proximal colon has rarely been reported.

**CASE PRESENTATION:**

A 55-year-old woman with a history of rectal cancer underwent multiple surgeries, including pelvic exenteration with a permanent end colostomy via an intraperitoneal route. Nine days after discharge following hepatectomy and inguinal lymph node dissection, she presented with abdominal pain and cessation of stool output. CT revealed obstruction at the stomal outlet. Decompression through the stoma using a drainage tube was attempted but failed. Emergency surgery was performed. Intraoperatively, no significant adhesions were observed. The proximal colon was markedly dilated and filled with feces. An elongated transverse colon and a mobilized splenic flexure had descended into the pelvis, and the fecal loading may have contributed to torsion of the stomal limb at the outlet. Reduction of the colon from the pelvis resolved the torsion. Fecal contents were evacuated via the stoma, and no bowel resection was required. The postoperative course was uneventful, and the patient was discharged on the 5th day after surgery. No recurrence was observed during the short-term follow-up.

**CONCLUSIONS:**

Torsion of a mobile stomal limb associated with fecal loading should be considered as a rare cause of stomal outlet obstruction, particularly when conservative decompression is ineffective. In patients with an elongated and mobile colon, appropriate bowel management may be important to prevent fecal retention and recurrence.

## Abbreviation

SOO
: stomal outlet obstruction

## INTRODUCTION

Stoma-related complications are relatively common after colorectal surgery and include obstruction, stenosis, prolapse, and parastomal hernia.^1,2)^ SOO is usually caused by adhesions, kinking, or stenosis at the abdominal wall.^3,4)^

In contrast, torsion of the stomal limb is an extremely rare mechanism, and its pathophysiology has not been well described. To the best of our knowledge, there are no reports of torsion of the stomal limb associated with fecal loading in the proximal colon. Herein, we report a rare case of SOO caused by torsion of an end colostomy limb associated with an elongated, feces-loaded colon.

## CASE PRESENTATION

**Fig. 1:**
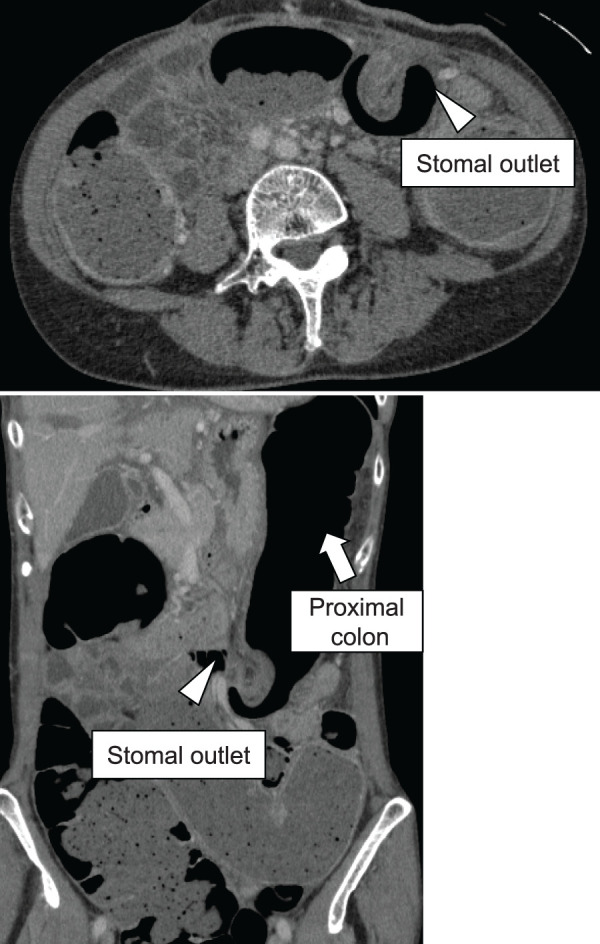
Contrast-enhanced CT showing stenosis at the stomal outlet (arrowhead) with marked dilatation of the proximal colon (arrow).

**Fig. 2:**
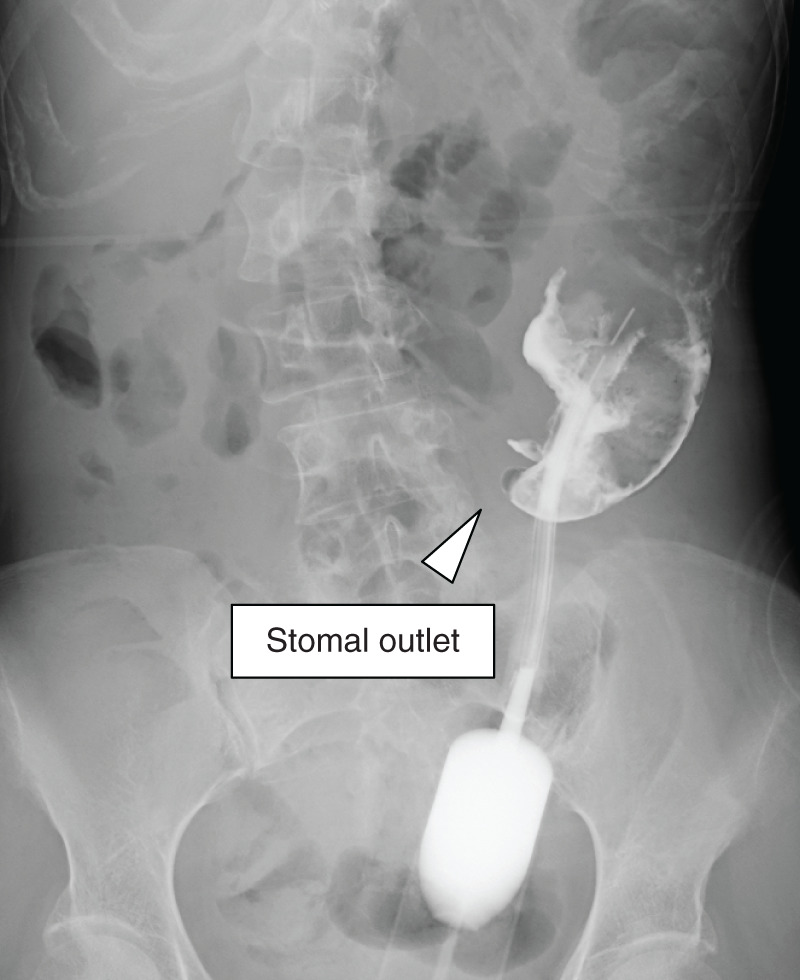
Fluoroscopic examination via a drainage tube inserted through the stoma demonstrating narrowing at the stomal outlet (arrowhead).

**Fig. 3:**
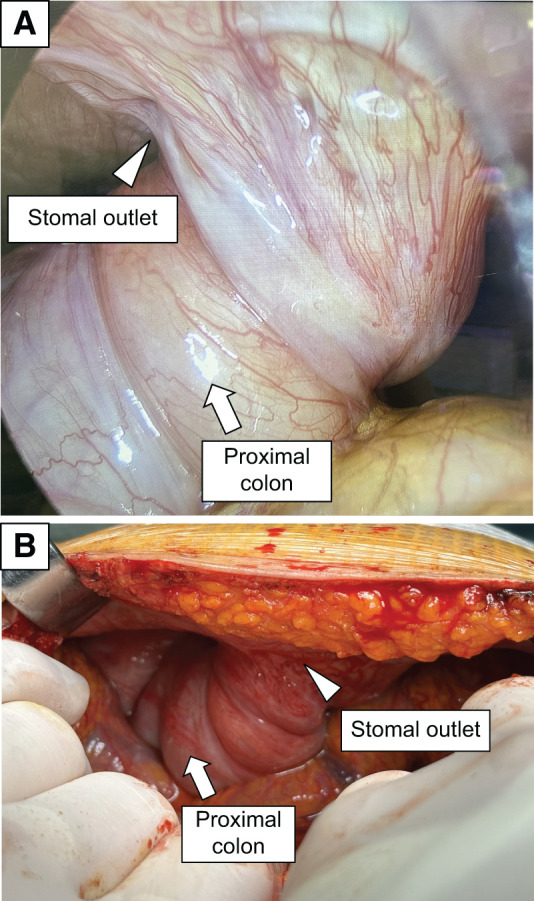
Intraoperative findings. (**A**) Laparoscopic findings showing torsion of the stomal limb at the outlet without significant intra-abdominal adhesions. (**B**) Laparotomy findings confirming torsion of the stomal limb near the stomal outlet. Stomal outlet (arrowhead) and proximal colon (arrow).

**Fig. 4:**
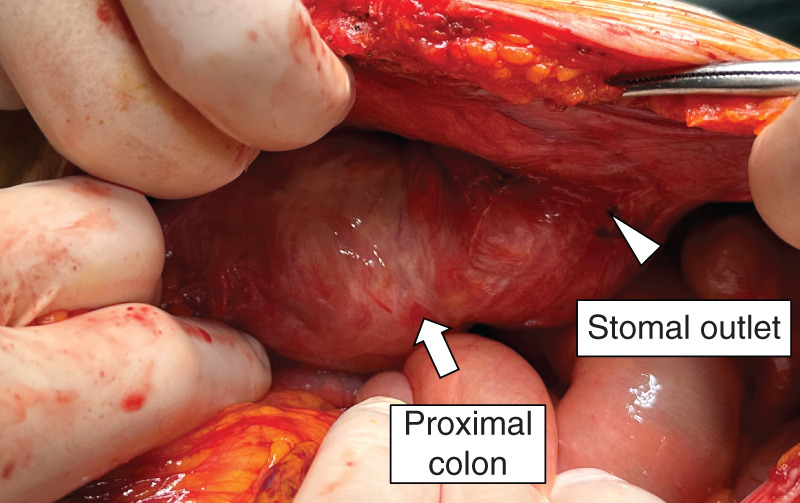
Intraoperative findings after reduction of the torsion. Stomal outlet (arrowhead) and proximal colon (arrow).

A 55-year-old woman with a history of advanced rectal cancer underwent multimodal treatment. Her surgical history included robot-assisted low anterior resection with splenic flexure mobilization, pulmonary resection for lung metastasis, hepatectomy for liver metastases, and laparoscopic posterior pelvic exenteration for local recurrence. A permanent end colostomy was created in the left lower abdomen via an intraperitoneal route. Nine days after discharge following her latest surgery, namely, robot-assisted hepatectomy for liver metastases and right inguinal lymph node dissection for inguinal lymph node metastasis, she presented with abdominal pain and cessation of flatus and stool. This occurred approximately 10 months after creation of the permanent end colostomy. Contrast-enhanced CT revealed stenosis at the stomal outlet and dilatation of the proximal colon with accumulation of gas and fecal contents (**Fig. 1**). A 10-Fr drainage tube was inserted through the stoma for decompression, and fluoroscopic examination demonstrated narrowing at the stomal outlet (**Fig. 2**). This tube size was selected because insertion of a larger-caliber tube through the narrowed stomal outlet was considered to increase the risk of mucosal injury or intestinal perforation. The purpose of tube placement was not complete evacuation of the solid fecal load, but rather gentle decompression of the dilated proximal colon and assessment of the response to conservative treatment. After tube insertion, stool discharge was obtained and radiographic findings showed improvement of colonic dilatation. Therefore, the tube was removed 3 days after the initial insertion. However, the patient’s abdominal symptoms recurred the following day. A 10-Fr tube was inserted again, but adequate decompression was not achieved and the abdominal symptoms tended to worsen. There were no obvious findings suggestive of intestinal ischemia or strangulation; however, the obstruction recurred shortly after tube removal, and repeated trans-stomal decompression was insufficient. Because persistent obstruction with marked fecal loading was considered to carry a risk of further colonic dilatation and deterioration of the patient’s general condition, continued conservative management or delayed elective surgery was considered inappropriate. Therefore, emergency surgery was performed 5 days after the initial tube insertion. Intraoperatively, laparoscopic exploration revealed no significant adhesions causing bowel obstruction. However, torsion of the stomal limb at the outlet was identified (**Fig. 3**). Due to marked bowel distension, conversion to laparotomy was required. The transverse colon had descended into the pelvis and was twisted near the stomal outlet. After the descended transverse colon was repositioned into the upper abdomen, the torsion was released (**Fig. 4**). After aspiration of the fecal contents as much as possible, a finger could be smoothly inserted through the stoma into the abdominal cavity, confirming that the obstruction and angulation had been resolved. These intraoperative findings suggested that the SOO was caused by torsion of the mobile stomal limb rather than simple kinking at the fascial level or local mucosal edema. The procedure was completed without bowel resection or stoma revision. The postoperative course was uneventful, and the patient was discharged on the 5th day after surgery. No recurrence was observed during 1 month of follow-up.

## DISCUSSION

**Fig. 5:**
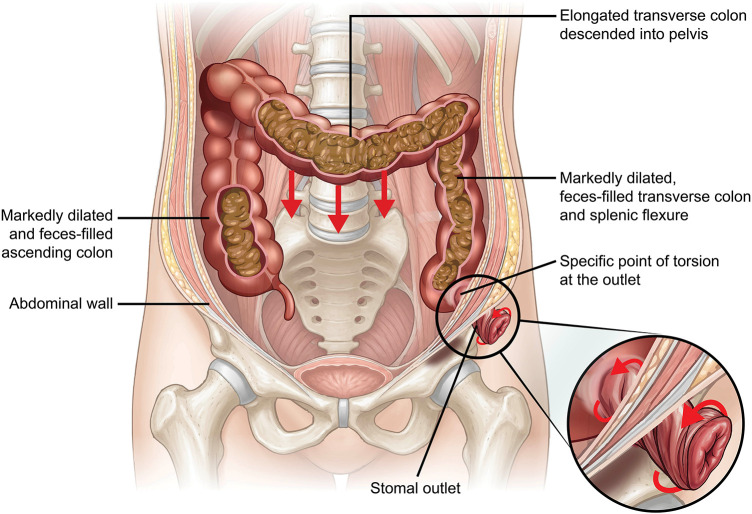
Schematic illustration of the inferred mechanism of SOO. Fecal loading in the elongated proximal colon may have contributed to descent of the mobile stomal limb into the pelvis and subsequent torsion near the stomal outlet. SOO, stomal outlet obstruction

With recent advances in perioperative treatment, the number of surgical interventions for advanced and recurrent colorectal cancer has increased, resulting in more frequent creation of temporary or permanent stomas.^5)^ Stoma-related complications are frequently encountered after colorectal surgery and include bowel obstruction, prolapse, stenosis, and parastomal hernia. Among these, bowel obstruction is a relatively common complication. The underlying causes of obstruction include adhesive ileus, SOO, and internal hernia through the space between the lifted bowel and the abdominal wall.^6,7)^ SOO is generally defined as an obstruction occurring at the level of the abdominal wall and is typically caused by kinking, edema, stenosis, or parastomal hernia.^3,4)^ In contrast, the present case demonstrated a different mechanism. The stomal limb was not obstructed by adhesions or stenosis but was instead twisted near the stomal outlet, possibly in association with fecal loading in the proximal colon. The elongated transverse colon and mobilized splenic flexure had descended into the pelvis, and this downward displacement may have exerted traction on the stomal limb, contributing to torsion at the stomal outlet (**Fig. 5**). However, this mechanism was inferred from the imaging and intraoperative findings and was not directly proven. To the best of our knowledge, there have been no previous reports describing SOO caused by torsion of the stomal limb associated with fecal loading. The key clinical message of this case is that torsion of a mobile stomal limb should be considered as a rare but important differential diagnosis in patients with unexplained SOO, particularly when conservative decompression using a trans-stomal tube is ineffective.

Several factors may have contributed to the development of torsion in the present case. First, the stoma had been created through an intraperitoneal route, which may have allowed greater mobility of the stomal limb. Second, the transverse colon was elongated and had descended into the pelvis. Third, previous mobilization of the splenic flexure may have further increased the mobility of the transverse colon. Finally, constipation and fecal retention may have increased the weight of the stomal limb, promoting its descent into the pelvis and subsequent torsion near the stomal outlet. However, because this is a single case, these factors should be interpreted as possible contributors rather than proven causes.

In the present case, only reduction of the torsion and evacuation of fecal contents were performed without fixation of the proximal colon or stoma reconstruction. This procedure relieved the acute obstruction but did not anatomically correct all possible predisposing factors, including the redundant colon, previous splenic flexure mobilization, and intraperitoneal stoma route. Colopexy was considered as a possible recurrence-preventive procedure; however, because the transverse colon and splenic flexure had been extensively mobilized, fixation under tension or at an inappropriate site was considered to carry a risk of bowel wall injury, internal herniation, or creation of a new torsion point. Reconstruction of the stoma via an extraperitoneal route was also considered, as this route has been reported to reduce postoperative complications such as bowel obstruction by covering and supporting the lifted bowel with the peritoneum, thereby limiting excessive mobility.^8,9)^ However, stoma reconstruction was considered relatively invasive in the emergency setting, particularly in the presence of marked colonic dilatation. Therefore, we selected reduction and fecal evacuation alone as the least invasive procedure to resolve the acute obstruction, while reserving colopexy or stoma reconstruction as possible options in case of recurrence. Postoperative bowel management, including the use of laxatives to prevent constipation and fecal retention, may be reasonable; however, its effectiveness in preventing recurrence of this condition cannot be concluded from this single case.

The present episode occurred within 1 month after hepatectomy and right inguinal lymph node dissection. Therefore, postoperative adhesions related to the recent surgeries were initially considered as a possible cause of obstruction. However, intraoperative findings showed no significant adhesions causing obstruction. In retrospect, the recent surgeries may have indirectly contributed to the development of SOO because laxatives had not been administered after hepatectomy, resulting in poor bowel control, constipation, and fecal retention. This complication occurred approximately 10 months after creation of the permanent end colostomy, suggesting that torsion of the stomal limb may develop not only in the early postoperative period but also in the late postoperative period when a mobile stomal limb, fecal retention, and poor bowel control coexist.

This case report has several limitations. First, this is a single case, and therefore the causal relationship between fecal loading and torsion of the stomal limb cannot be definitively established. Second, the follow-up period was short. Although no recurrence was observed during the short follow-up period, longer follow-up is necessary to determine whether reduction alone combined with postoperative bowel management is sufficient to prevent recurrence. Third, the effectiveness of bowel management in preventing recurrence remains unproven. Bowel distension may occur not only because of constipation and fecal retention but also because of other conditions, such as paralytic ileus or chronic intestinal pseudo-obstruction. Therefore, recurrence through a similar mechanism remains possible even with appropriate bowel management. Fourth, the optimal surgical strategy for this condition remains unclear, including whether fixation of the proximal colon or reconstruction of the stoma via an extraperitoneal route should be performed. Although an extraperitoneal route may theoretically reduce the mobility of the stomal limb and could be considered as a preventive option, no firm recommendation can be made based on a single case. Further accumulation of similar cases is necessary to clarify the optimal stoma route and preventive strategy.

## CONCLUSIONS

Torsion of a mobile stomal limb should be considered as a rare but important cause of SOO, particularly in patients with an elongated, feces-loaded colon and excessive colonic mobility. Because the optimal strategy to prevent recurrence remains unclear, careful long-term follow-up is required.

## DECLARATIONS

### Availability of data and materials

All data generated or analyzed during this study are included in this published article.
